# Regional and racial disparity in proximal gastric cancer survival outcomes 1996–2016: Results from SEER and China National Cancer Center database

**DOI:** 10.1002/cam4.4033

**Published:** 2021-06-09

**Authors:** Lulu Zhao, Penghui Niu, Dongbing Zhao, Yingtai Chen

**Affiliations:** ^1^ Department of Pancreatic and Gastric Surgical Oncology National Cancer Center/National Clinical Research Center for Cancer/Cancer Hospital, Chinese Academy of Medical Sciences and Peking Union Medical College Beijing China

**Keywords:** proximal gastric cancer, racial disparity, regional disparity, survival outcomes

## Abstract

**Background:**

Given the growing incidence and aggressive biological behavior of proximal gastric cancer (PGC) as reported, it is important to understand which regional or racial populations are at poor prognosis so that interventions can be treated appropriately. We sought to explore regional treatment differences as well as racial genes influence survival outcomes in China and the US patients with PGC.

**Methods:**

PGC patients defined as tumors with the epicenter located in cardia (C16.0) or fundus (C16.1) from 1996 to 2016 were identified from the Surveillance Epidemiology and End Results (SEER) in the United States as well as data from a high‐volume National Cancer Center Database in China. Overall survival (OS) curves were plotted for different regional or racial groups, respectively, using the Kaplan‐Meier method and compared statistically using the log‐rank test. Differentially expressed genes (DEGs) analysis was performed using TCGA database.

**Results:**

Finally, the cohort consistent of 40973 PGC patients who enrolled in SEER database (n = 36305) or China National Cancer Center (n = 4668), and divided into 4 racial groups: Chinese (n = 5179), Black (n = 2429), White (n = 31185), and Others (n = 2096). After controlling for confounding variables, racial factors were independently associated with poor survival included Black ethnicity (HR = 1.376, 95% CI: 1.066–1.7760, *p* = 0.014) and White ethnicity (HR = 1.262, 95% CI: 1.005–1.583, *p* = 0.045) when compared to Chinese ethnicity in total PGC patients. Even in the same region for only US group, Chinese PGC patients also showed better prognosis.

**Conclusions:**

In conclusion, we demonstrated the different survival outcomes of PGC patients in different regions or races from two high‐volume database SEER and China National Cancer Center database. These survival differences are likely influenced by a number of factors (e.g., access to screening, quality of gastrectomy, neo/adjuvant therapy, and biological genes itself). More importantly, a better understanding of these disparities could lead to interventions that may help to abolish these disparities.

## Graphical abstract

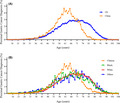

## 1 INTRODUCTION

Gastric cancer is the third leading cause of cancer‐related mortality and the fifth most common cancer globally.^1^ Notably, there are differences in incidence, prevalence and mortality of gastric cancer in different regions or races.^2^, ^3^, ^4^, ^5^, ^6^, ^7^, ^8^ For example, notwithstanding the higher prevalence of gastric cancer in Asia, significantly better outcomes have been reported in Asia compared to Western counties.^9^ In fact, important differences have also been observed in gastric cancer presentation, anatomic location (proximal‐cardia, fundus; distal‐body, antrum, pylorus) and patients receipt of multi‐modality therapy and surgery.^2^, ^3^, ^4^, ^5^, ^6^, ^7^, ^8^

Anatomic differences in location of gastric cancer between Western and Asian nations may contribute to such differences with proximal gastric cancer (PGC) being more prevalent in Western countries compared to distal gastric cancer (DGC) being more prevalent in Asian countries.^10^, ^11^, ^12^ Given the growing incidence and aggressive biological behavior of PGC as reported,^12^ it is important to understand which subpopulations are at worst prognosis of dying from each so that interventions can be treated appropriately. Amy et al^13^ utilized the California Cancer Registry showed no significant difference in survival with respect to race in cardia gastric cancer, while better survival in Asians than other races was seen in some studies of gastric cancer.^5^, ^14^, ^15^, ^16^, ^17^ In summary, the clinicopathological features and survival outcomes in different regions or races of PGC patients are ambiguous, which included not only cardia but also fundus cancers of stomach.

As such, utilizing a unique combination of the Surveillance Epidemiology and End Results (SEER) in the US as well as data from a high‐volume National Cancer Center Database in China, we sought to explore the extent to regional treatment differences as well as racial genes influence survival outcomes in China and the US patients with PGC.

We present the following article in accordance with the STROBE reporting checklist.

## 2 METHODS

### 2.1 Study subjects

This study were abstracted from SEER 18 Regs Custom Data (with additional treatment fields) Nov 2018 Sub (1975–2016 varing), and also the China National Cancer Center. The China National Cancer Center Database was a clinical gastric cancer database based on a huge retrospective cohort, and included more than 19,000 gastric cancer patients from all around China from 1997 to 2019. PGC was defined as tumors with the epicenter located in cardia (C16.0) or fundus (C16.1) in our research. In other word, PGC was considered to be esophagastric junction cancers (Siewert‐Stein type II and III) or fundus cancers. In total, 40973 PGC patients diagnosed in 1996–2016 year with certain region and race were included. Patients were categorized by region into 2 groups: China and the US, and by race into 4 groups: Chinese, White, Black, and Others. All staging data within this study were updated and coded to confirm to the American Joint Committee on Cancer (AJCC) TNM 7th edition staging system. T stage, categorized as T1, T2, T3, and T4; N stage, categorized as N0, N1, N2, and N3; M stage, categorized as M0 and M1, were determined by AJCC TNM 7th edition.

### 2.2 Statistical analysis

Categorical variables were compared using the chi‐squared test and continuous variables were analyzed by Student's *t*‐test. Overall survival (OS) curves were plotted for different regional or racial groups, respectively, using the Kaplan‐Meier method and compared statistically using the log‐rank test. Hazard ratios (HRs) and 95% confidence intervals (CIs) were used to estimate the risk of death by employing the multivariate Cox proportional hazards models with adjustment for region, race, age, sex, year, grade, linits plastica, signet ring cell carcinoma, AJCC TNM 7th ed, surgery, lymphadenectomy with at least 15 lymph nodes, neo/adjuvant chemotherapy, and neo/adjuvant radiation. Neo/adjuvant chemotherapy means neoadjuvant chemotherapy or/and adjuvant chemotherapy, while neo/adjuvant radiation means neoadjuvant radiation or/and adjuvant radiation. Variables with p values less than 0.10 on univariate analysis were subjected to the multivariate Cox regression model. Statistical analyses were performed using SPSS version 26.0 (College Station, TX, USA). A 2‐tailed P value less than 0.05 was considered statistically significant for all the tests.

### 2.3 Differentially Expressed GENES (DEGS) analysis from TCGA database

The R program package limma v3.28.14 (https://www.bioconductor.org/packages/devel/bioc/vignettes/limma/inst/doc/users‐guide.pdf) was used to analyze gene expression data for Asian and White gastric cancer patients. The mRNAs satisfying *p *< 0.01, false discovery rate (FDR) <0.01, and |log2 fold change (FC)|>log2 (1.5) were further investigated, where adjust *p *< 0.05 indicates that the hypothesis test was statistically significant and FDR is a control index for the hypothesis test error rate. As an evaluation index of the selected differential genes, the number of false rejections was proportional to the number of rejected null hypotheses. FC is generally used to describe the degree of change from an initial to a final value. Volcano diagram of the differential genes were constructed in R (https://cran.r‐project.org/web/packages/pheatmap/pheatmap.pdf) for easy visual comparison.

## 3 RESULTS

### 3.1 Descriptive statistics

**FIGURE 1:**
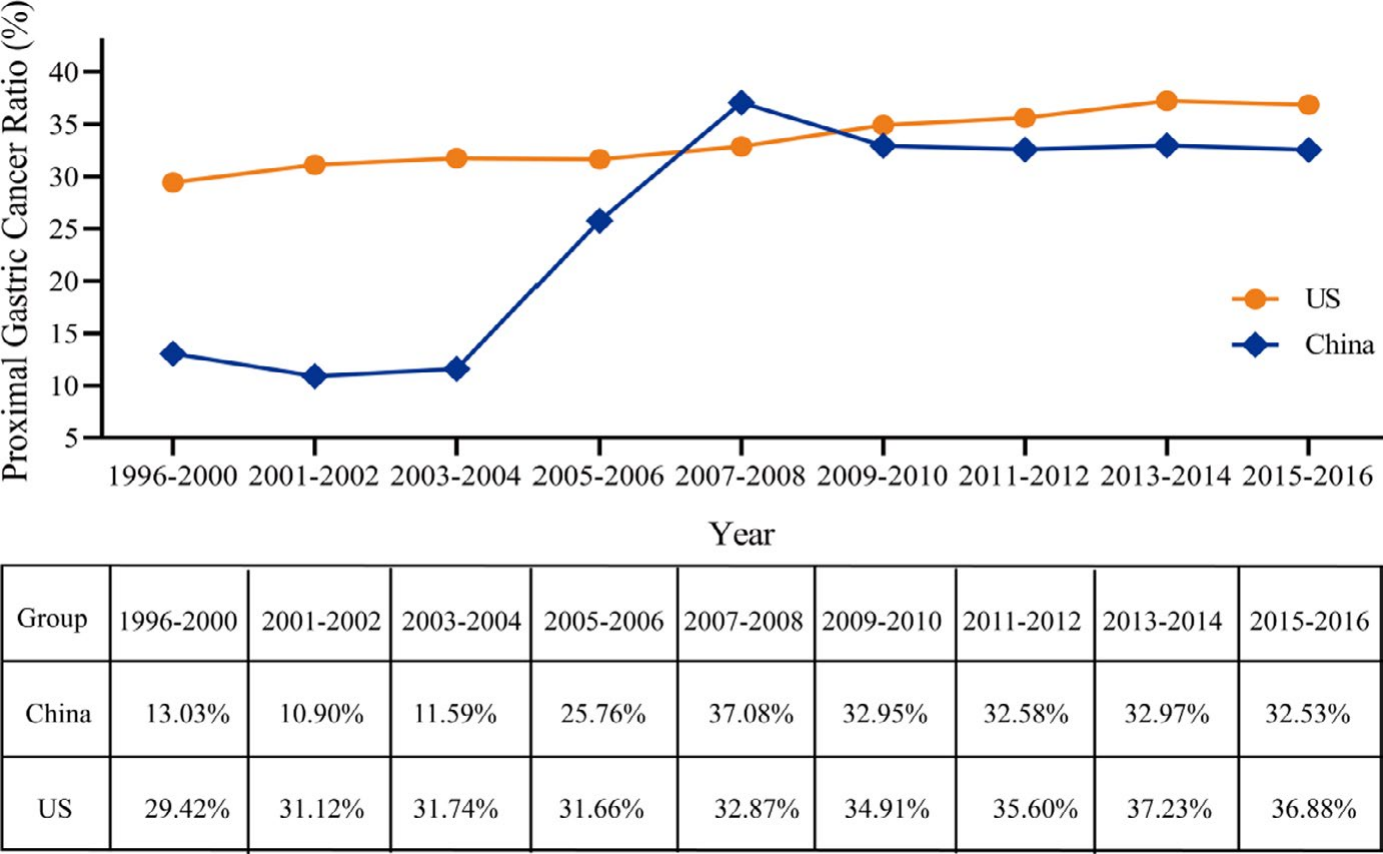
Ratio of proximal gastric cancer from SEER database and China National Cancer Center database among different years

**FIGURE 2:**
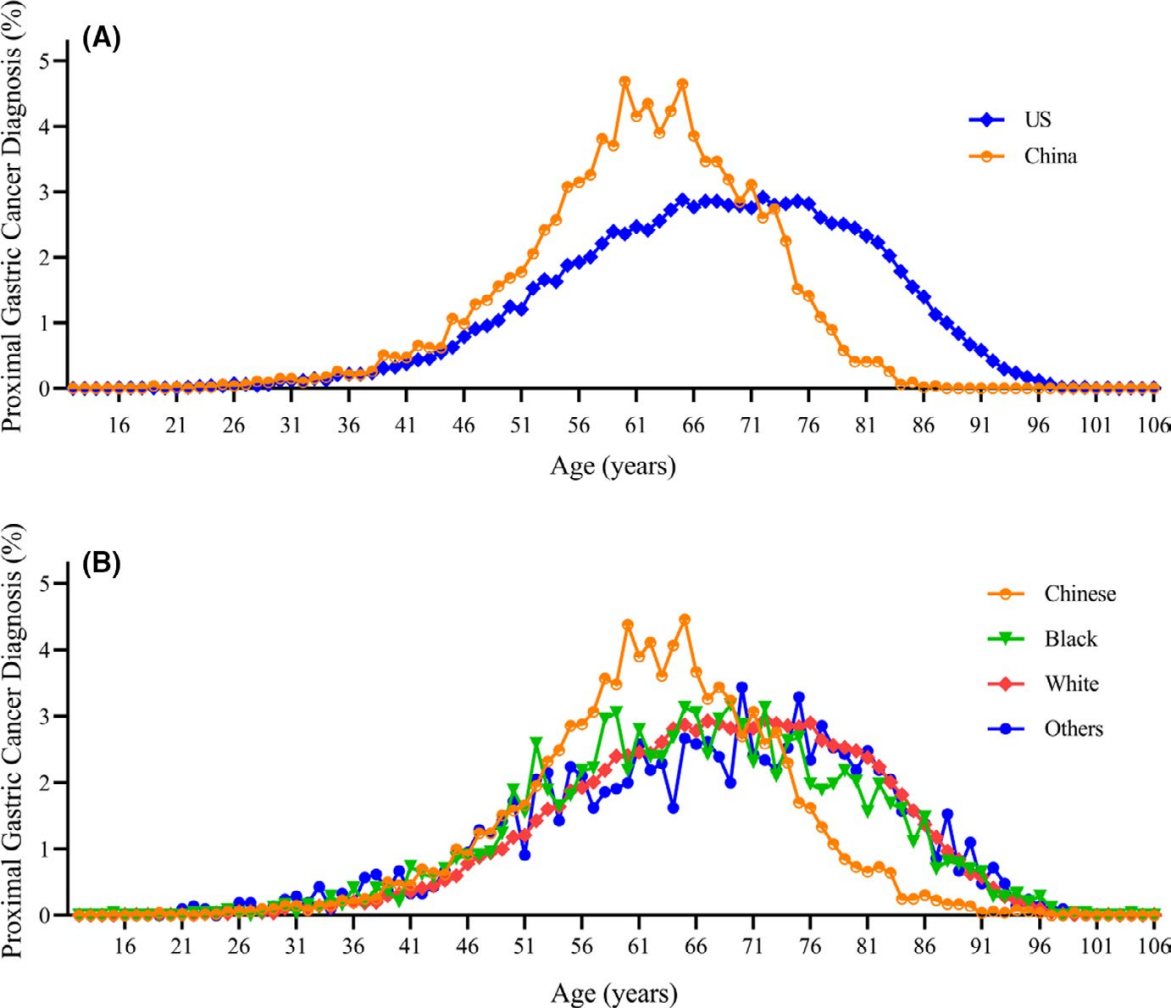
Diagnosis of proximal gastric cancer by patient age in different (A) regions and (B) races

As showed in Figure 1, the ratio of PGC patients from1996 to 2016 was rising in SEER database and China National Cancer Center. Although SEER database showed a higher ratio of PGC patients in total gastric cancer patients, a more significant growth was seen in China National Cancer Center. Finally, the cohort consistent of 40973 PGC patients who enrolled in SEER database (n = 36305) or China National Cancer Center (n = 4668). As outlined in Table 1, these patients included 4 races: Chinese (n = 5179), Black (n = 2429), White (n = 31185), and Others (n = 2096). When compared with PGC patients in the US, those identified in China were more likely at diagnosed at a younger age (61.28±9.877 vs. 67.59±12.962, *p *< 0.001) (Figure 2A,B).

**TABLE 1:** The clinicopathological features of proximal gastric cancer between China and the US in 1996–2016

	the US		China		Total		*p* value
	n	%	n	%	n	%	
Total	36305	100.0	4668	100.0	40973	100.0	
Age							
<45	1614	4.4	256	5.5	1870	4.6	<0.001
45–54	4215	11.6	783	16.8	4998	12.2	
55–64	8334	23.0	1790	38.3	10124	24.7	
65–74	10265	28.3	1503	32.2	11768	28.7	
>=75	11877	32.7	336	7.2	12213	29.8	
Sex							
Female	8772	24.2	751	16.1	9523	23.2	<0.001
Male	27533	75.8	3917	83.9	31450	76.8	
Race							
Chinese	511	1.4	4668	100.0	5179	12.7	<0.001
Black	2429	6.7	0	0.0	2429	5.9	
White	31185	86.1	0	0.0	31185	76.3	
Others	2096	5.8	0	0.0	2096	5.1	
Year							
1996–2000	5213	14.4	80	1.7	5293	12.9	<0.001
2001–2005	8794	24.2	453	9.7	9247	22.6	
2006–2010	9481	26.1	1520	32.6	11001	26.8	
2011–2016	12817	35.3	2615	56.0	15432	37.7	
Grade							
Well differentiated	1599	5.3	147	4.3	1746	5.2	<0.001
Moderately differentiated	10045	33.0	1060	30.7	11105	32.8	
Poorly differentiated	18059	59.4	2212	64.0	20271	59.8	
Undifferentiated	712	2.3	37	1.1	749	2.2	
Linits plastica							
Yes	118	0.3	24	0.5	142	0.3	0.038
No/unknown	36187	99.7	4644	99.5	40831	99.7	
Signet ring cell carcinoma							
Yes	4019	11.1	667	14.3	4686	11.4	<0.001
No/unknown	32286	88.9	4001	85.7	36287	88.6	
AJCC T,7^th^ed							
T1	6331	35.4	486	12.8	6817	31.5	<0.001
T2	4045	22.6	332	8.7	4377	20.2	
T3	5266	29.5	1446	38.0	6712	31.0	
T4	2223	12.4	1538	40.5	3761	17.4	
AJCC N,7^th^ed							
N0	9814	48.2	1229	32.9	11043	45.9	<0.001
N1	8051	39.6	733	19.6	8784	36.5	
N2	1697	8.3	792	21.2	2471	10.3	
N3	796	3.9	987	26.4	1783	7.4	
AJCC M,7^th^ed							
M0	14619	64.1	4009	90.3	18628	68.4	<0.001
M1	8179	35.9	429	9.7	8608	31.6	
AJCC TNM, 7^th^ed							
I	5140	24.4	653	16.0	5793	23.1	<0.001
II	4137	19.7	900	22.1	5037	20.1	
III	3584	17.0	2098	51.4	5682	22.6	
IV	8179	38.9	429	10.5	8608	34.3	
Surgery							
Yes	14342	39.5	3796	81.3	18138	44.3	<0.001
No	21375	58.9	872	18.7	22243	54.3	
Unknown	592	1.6	0	0.0	592	1.4	
Lymphadenectomy with at least 15 lymph nodes							
Yes	5315	35.6	2670	70.3	7985	42.6	<0.001
No/unknown	9619	64.4	1126	29.7	10745	57.4	
Number positive nodes							
Yes	6580	44.1	2355	62.0	8935	47.7	<0.001
No/unknown	8354	55.9	1441	38.0	9795	52.3	
Neo/adjuvant Chemotherapy^a^							
Yes	7439	51.9	1314	34.6	8753	48.3	<0.001
No/unknown	6903	48.1	2482	65.4	9385	51.7	
Neo/adjuvant Radiation^a^							
Yes	5796	40.4	173	4.6	5969	32.9	<0.001
No/unknown	8546	59.6	3623	95.4	12169	67.1	
Age (year)							
Mean	67.59		61.28		66.87		<0.001
SD	12.962		9.887		12.807		
Number nodes examined (n)							
Mean	13.03		23.10		15.02		
SD	11.764		12.008		12.473		0.911
Number positive nodes (n)							
Mean	3.18		5.12		3.62		
SD	5.375		7.083		5.858		<0.001

Neo/adjuvant Radiation: Neoadjuvant radiation OR/AND adjuvant radiation.

^a^ Neo/adjuvant Chemotherapy: Neoadjuvant chemotherapy OR/AND adjuvant chemotherapy.

There are notable differences of the entire cohort between China and the US in basic clinicopathological features (Table 1). Compared to the US group, PGC patients in China showed a more percentage of poorly differentiated (64.0% vs. 59.4%, *p *< 0.001), Linits plastica (0.5% vs. 0.3%, *p* = 0.038) and Signet ring cell carcinoma (14.3% vs. 11.1%, *p *< 0.001). As for TNM stage, China group were more likely to be in later T stage (T4, 40.5% vs. 12.4%, *p *< 0.001) and N stage (N3, 26.4% vs. 3.9%, *p *< 0.001), but less distant metastasis (M1, 9.7% vs. 35.9, *p *< 0.001). Additionally, the US patients were much more likely to have later TNM stage IV tumors (38.9% vs. 10.5%, *p *< 0.001) than China group. A higher proportion of gastrectomy (81.3% vs. 39.5%, *p *< 0.001) and adequate lymphadenectomy with at least 15 lymph nodes (70.3% vs. 35.6%, *p *< 0.001) was performed in China group of PGC patients compared to the US patients. Not surprising, more Neo/adjuvant Chemotherapy and Radiation were performed in the US PGC patients than those in China group.

### 3.2 Unadjusted and adjusted survival analysis in different regions

**FIGURE 3:**
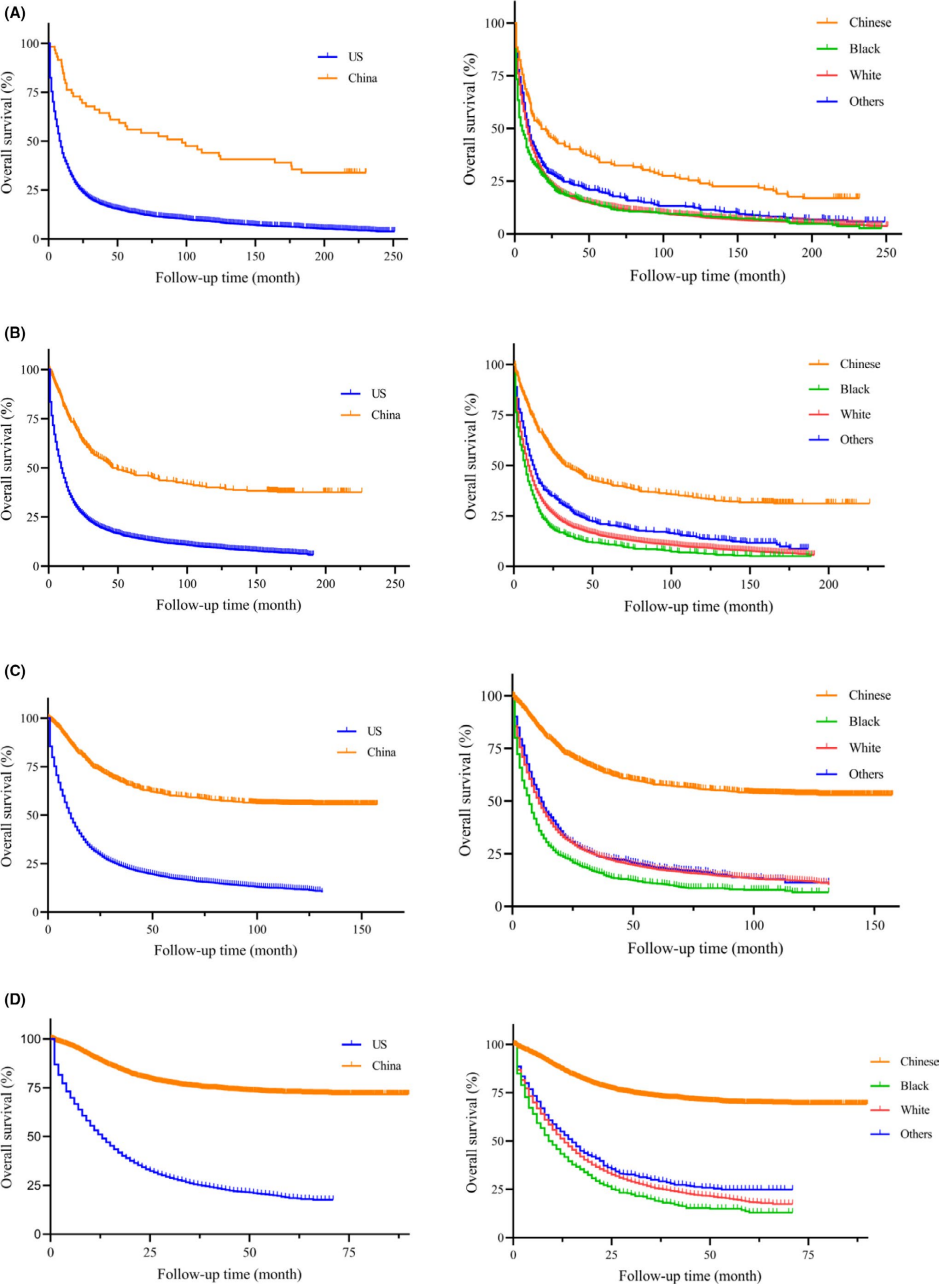
Kaplan‐Meier survival curves of different races and regions from China National Cancer Center database and SEER database in (A) 1996–2000 year, (B) 2001–2005 year, (C) 2006–2010 year, (D) 2011–2016 year

**FIGURE 4:**
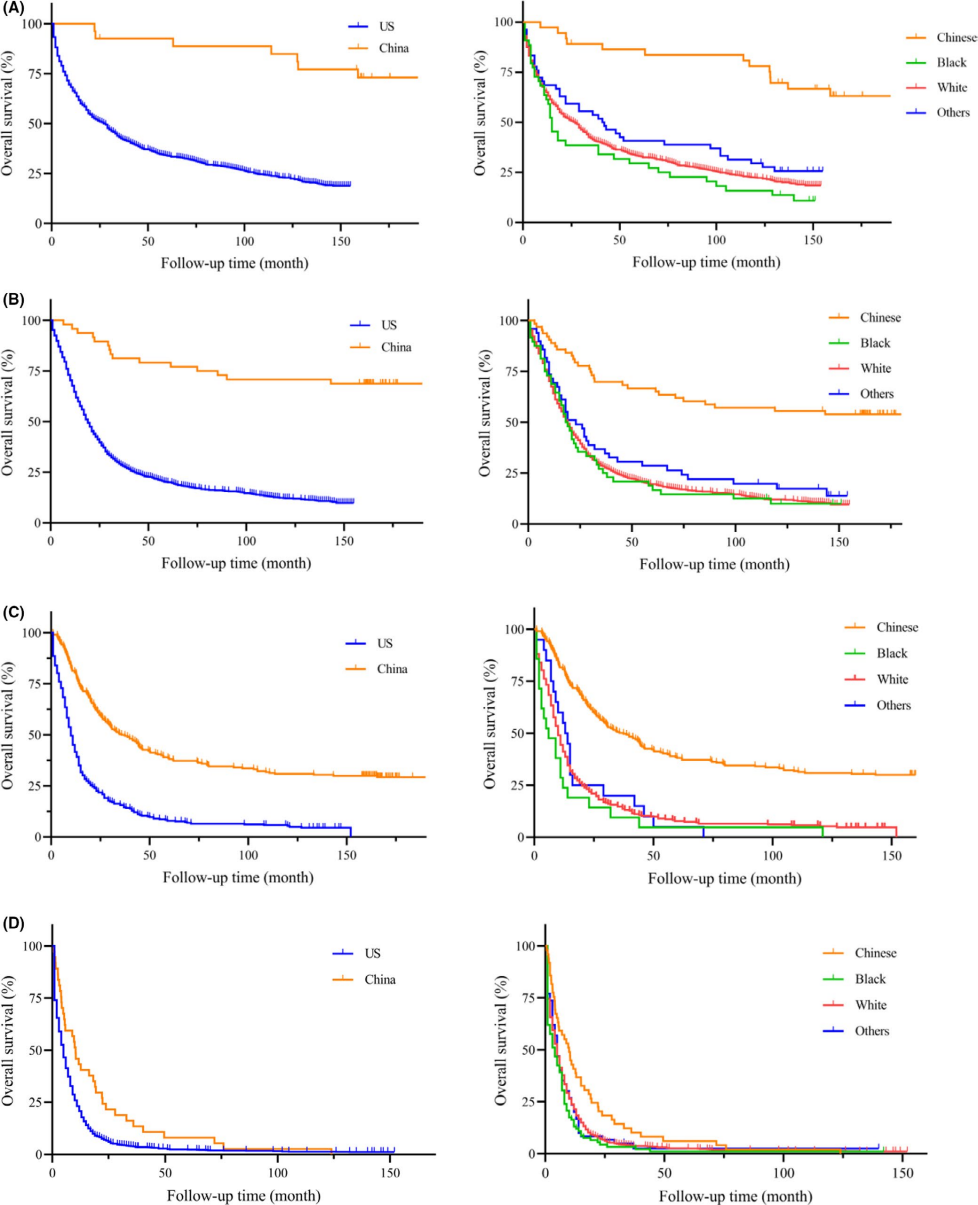
Kaplan‐Meier survival curves of different regions and races from China National Cancer Center database and SEER database in 2001–2005 year diagnosed as TNM stage (A) I, (B) II, (C) III, (D) IV

**FIGURE 5:**
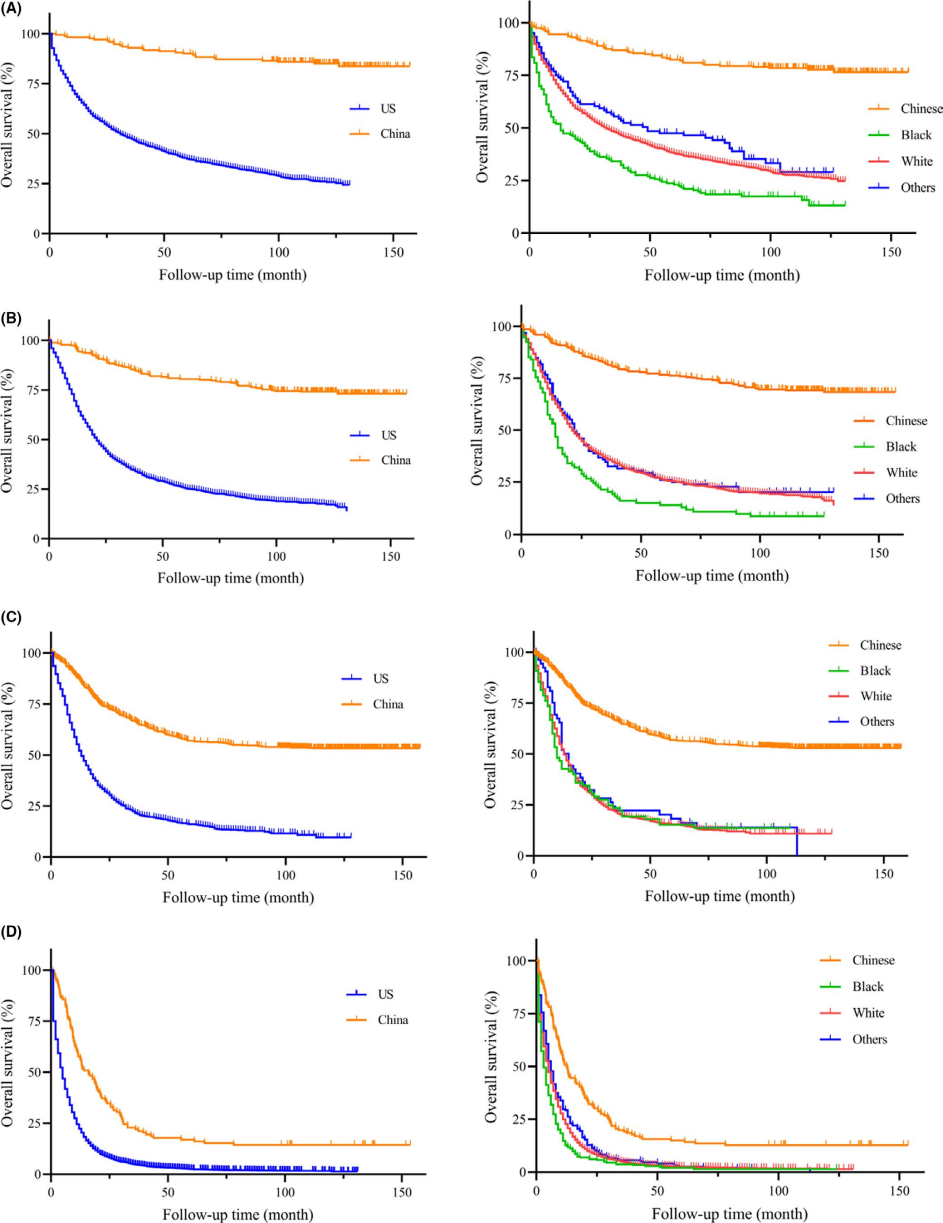
Kaplan‐Meier survival curves of different regions and races from China National Cancer Center database and SEER database in 2006–2010 year diagnosed as TNM stage (A) I, (B) II, (C) III, (D) IV

On unadjusted analysis (Table 2), China group had a better prognosis when compared to the US patients (HR = 4.337, 95% CI: 4.123–4.562, *p *< 0.001). To avoid the bias of time, the Kaplan‐Meier survival curves of PGC patients between China and the US were presented with different year period (Figure 3, left column). Similarly, PGC patients in China group had a significantly longer median survival than the US patients (all *p *< 0.001). In our further analysis, we performed detailed Kaplan‐Meier survival analysis of PGC patients. Figure 4 (left column) showed the Kaplan‐Meier survival curves of PGC patients between China and the US in 2001–2005 year diagnosed as TNM stage I (Figure 4A), II (Figure 4B), III (Figure 4C), IV (Figure 4D), while Figure 5 (left column) in 2006–2010, Figure 6 (left column) in 2010–2016. All these figures showed obvious survival benefit of PGC patients who diagnosed in China (all *p *> 0.001).

**TABLE 2:** Unadjusted survival analysis of proximal gastric cancer by different regions and races

	Total		*p* value	the US		*p* value	China		*p* value
	HR	95%CI		HR	95%CI		HR	95%CI	
Region									
China	1			‐			‐		
the US	4.337	4.123–4.562	<0.001						
Race									
Chinese	1			1			‐		
Black	4.538	4.266–4.827	<0.001	1.431	1.284–1.596	<0.001			
White	3.716	3.549–3.890	<0.001	1.173	1.173–1.298	0.002			
Others	3.28	3.071–3.504	<0.001	1.036	0.927–1.158	0.533			
Age									
<45	1			1			1		
45–54	0.936	0.878–0.997	0.039	0.993	0.930–1.061	0.843	0.689	0.556–0.854	0.001
55–64	0.93	0.877–0.986	0.015	1.049	0.987–1.116	0.126	0.553	0.453–0.675	<0.001
65–74	1.088	1.027–1.152	0.004	1.160	1.092–1.232	<0.001	0.638	0.522–0.779	<0.001
>=75	1.766	1.668–1.869	<0.001	1.675	1.578–1.778	<0.001	0.836	0.654–1.070	0.154
Sex									
Female	1			1			1		
Male	0.908	0.885–0.932	<0.001	0.963	0.937–0.989	0.005	0.958	0.839–1.093	0.519
Year									
1996–2000	1			1			1		
2001–2005	0.919	0.887–0.952	<0.001	0.952	0.919–0.987	0.007	1.034	0.737–1.451	0.845
2006–2010	0.731	0.706–0.758	<0.001	0.863	0.832–0.894	<0.001	0.679	0.491–0.939	0.019
2011–2016	0.61	0.589–0.631	<0.001	0.788	0.760–0.817	<0.001	0.447	0.323–0.618	<0.001
Grade									
Well differentiated	1			1			1		
Moderately differentiated	1.307	1.227–1.392	<0.001	1.333	1.251–1.421	<0.001	3.686	1.959–6.934	<0.001
Poorly differentiated	1.693	1.592–1.800	<0.001	1.800	1.692–1.914	<0.001	5.599	2.999–10.451	<0.001
Undifferentiated	2.071	1.877–2.285	<0.001	1.859	1.683–2.053	<0.001	30.572	13.729–68.077	<0.001
Linits plastica									
Yes	1			1			1		
No/unknown	0.69	0.580–0.822	<0.001	0.658	0.547–0.791	<0.001	0.421	0.249–0.713	0.001
Signet ring cell carcinoma									
Yes	1			1			1		
No/unknown	0.923	0.892–0.956	<0.001	0.857	0.827–0.888	<0.001	0.986	0.858–1.132	0.836
AJCC T,7^th^ed									
T1	1			1			1		
T2	0.931	0.890–0.975	0.002	0.915	0.874–0.958	<0.001	1.585	1.025–2.451	0.038
T3	0.871	0.835–0.907	<0.001	1.034	0.990–1.079	0.130	3.792	2.728–5.270	<0.001
T4	1.108	1.056–1.162	<0.001	2.033	1.928–2.144	<0.001	6.708	4.856–9.265	<0.001
AJCC N,7^th^ed									
N0	1			1			1		
N1	1.303	1.261–1.347	<0.001	1.255	1.213–1.297	<0.001	1.938	1.574–2.386	<0.001
N2	0.89	0.843–0.939	<0.001	1.115	1.052–1.182	<0.001	2.835	2.339–3.437	<0.001
N3	0.93	0.874–0.989	0.022	1.407	1.301–1.523	<0.001	5.029	4.220–5.993	<0.001
AJCC M,7^th^ed									
M0	1			1			1		
M1	3.401	3.301–3.505	<0.001	2.838	2.752–2.927	<0.001	4.831	4.219–5.530	<0.001
AJCC TNM, 7^th^ed									
I	1			1			1		
II	1.168	1.113–1.226	<0.001	1.296	1.234–1.362	<0.001	2.336	1.699–3.212	<0.001
III	1.206	1.149–1.265	<0.001	1.579	1.499–1.664	<0.001	6.559	4.931–8.724	<0.001
IV	4.255	4.078–4.4438	<0.001	3.942	3.776–4.116	<0.001	22.752	16.790–30.830	<0.001
Surgery									
Yes	1			1			1		
No	3.588	3.501–3.678	<0.001	3.198	3.116–3.281	<0.001	2.915	2.619–3.245	<0.001
Unknown	2.33	2.114–2.567	<0.001	1.941	1.761–2.139	<0.001			
Lymphadenectomy with at least 15 lymph nodes									
Yes	1			1			1		
No/unknown	1.469	1.414–1.527	<0.001	1.186	1.138–1.237	<0.001	1.162	1.025–1.316	0.019
Number positive nodes									
Yes	1			1			1		
No/unknown	0.616	0.593–0.639	<0.001	0.528	0.507–0.549	<0.001	0.420	0.365–0.483	<0.001
Neo/adjuvant Chemotherapy^a^									
Yes	1			1			1		
No/unknown	0.871	0.838–0.904	<0.001	0.990	0.951–1.031	0.633	0.888	0.785–1.003	0.057
Neo/adjuvant Radiation^a^									
Yes	1			1			1		
No/unknown	0.782	0.752–0.814	<0.001	1.038	0.997–1.082	0.070	0.761	0.580–0.998	0.048

^a^Neo/adjuvant Chemotherapy: Neoadjuvant chemotherapy OR/AND adjuvant chemotherapy.

Neo/adjuvant Radiation: Neoadjuvant radiation OR/AND adjuvant radiation.

**FIGURE 6:**
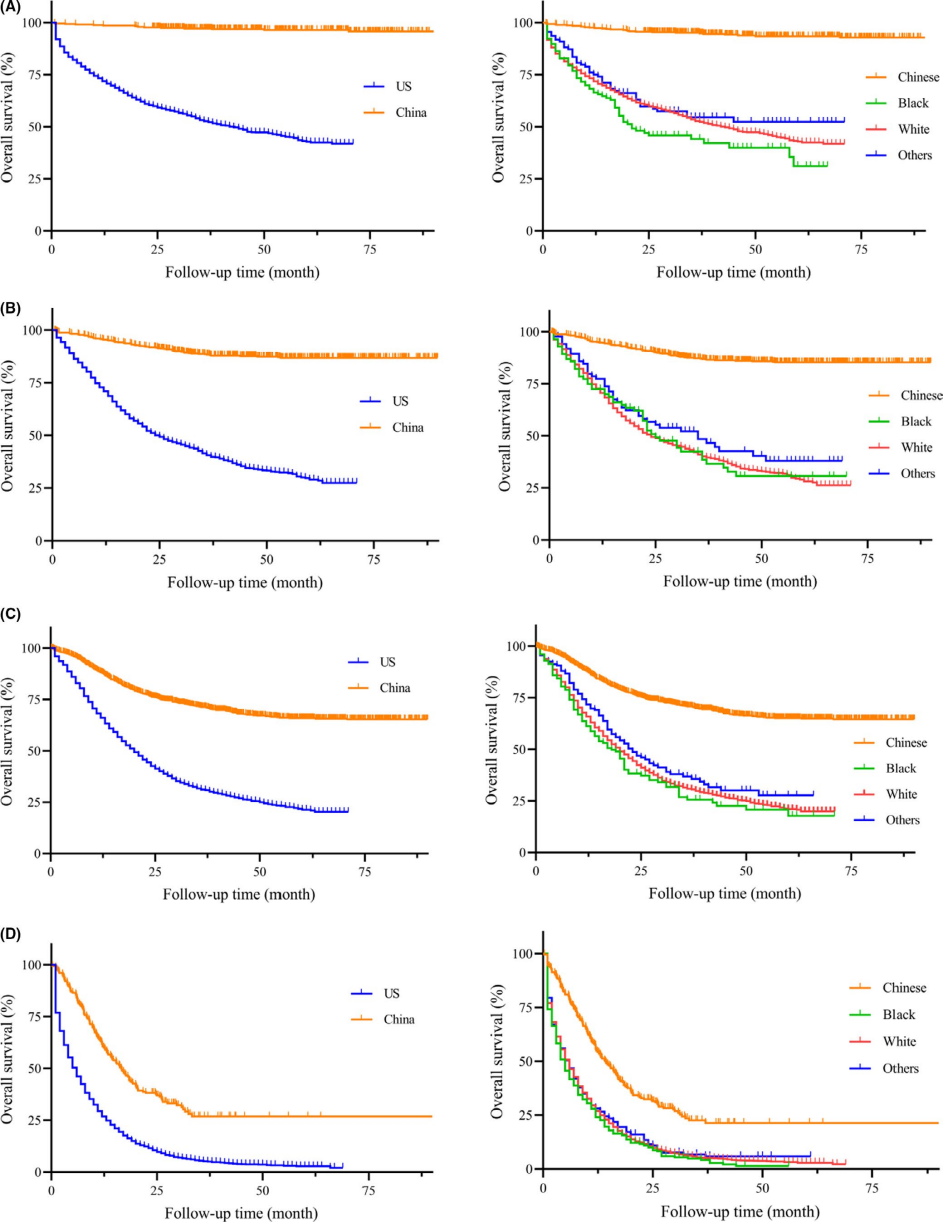
Kaplan‐Meier survival curves of different regions and races from China National Cancer Center database and SEER database in 2011–2016 year diagnosed as TNM stage (A) I, (B) II, (C) III, (D) IV

When appropriate significant factors were taken into consideration, multivariate analysis (Table 3) also revealed that the US group was an independent predictor for poor prognosis of PGC patients (HR = 4.137, 95% CI: 3.757–4.788, *p *< 0.001). When compared to young patients (<45year), middle year group (55–64 year) was associated with poor survival outcomes in the US (HR = 1.267, 95% CI: 1.089–1.473, *p* = 0.002) but favorable survival in China (HR = 0.705, 95% CI: 0.529–0.941, *p* = 0.018). Additional factors associated with increased survival in total PGC patients included adequate lymphadenectomy with at least 15 lymph nodes (HR = 1.265, 95% CI: 1.200–1.334, *p *< 0.001), neo/adjuvant chemotherapy (HR = 1.056, 95% CI: 1.036–1.433, *p* = 0.013) and neo/adjuvant radiation (HR = 1.207, 95% CI: 1.122–1.297, *p *< 0.001).

**TABLE 3:** Adjusted survival analysis of proximal gastric cancer by different regions and races

	Total		*p* value	the US		*p* value	China		*p* value
	HR	95%CI		HR	95%CI		HR	95%CI	
Region									
China	1			‐			‐		
the US	4.137	3.575–4.788	<0.001						
Race									
Chinese	1			1			‐		
Black	1.376	1.066–1.776	0.014	1.403	1.087–1.811	0.009			
White	1.262	1.005–1.583	0.045	1.274	1.016–1.599	0.036			
Others	1.034	0.803–1.332	0.796	1.044	0.811–1.345	0.738			
Age									
<45	1			1			1		
45–54	1.062	0.920–1.225	0.411	1.097	0.933–1.290	0.264	0.841	0.619–1.141	0.266
55–64	1.141	0.998–1.304	0.053	1.267	1.089–1.473	0.002	0.705	0.529–0.941	0.018
65–74	1.377	1.206–1.572	<0.001	1.527	1.314–1.773	<0.001	0.841	0.629–1.125	0.244
>=75	2.069	1.804–2.373	<0.001	2.284	1.960–2.662	<0.001	1.169	0.822–1.664	0.385
Sex									
Female	1			1			1		
Male	1.044	0.980–1.112	0.184	1.057	0.988–1.132	0.110	0.999	0.841–1.187	0.995
Year									
1996–2000	1			1			1		
2001–2005	0.901	0.611–1.330	0.601	0.905	0.703–1.302	0.372	0.997	0.661–1.502	0.987
2006–2010	0.717	0.487–1.055	0.092	0.845	0.786–0.908	<0.001	0.622	0.418–0.924	0.019
2011–2016	0.510	0.346–0.751	0.001	0.597	0.550–0.647	<0.001	0.472	0.316–0.703	<0.001
Grade									
Well differentiated	1			1			1		
Moderately differentiated	1.181	1.039–1.343	0.011	1.149	1.008–1.311	0.038	2.262	1.159–4.413	0.017
Poorly differentiated	1.544	1.360–1.753	<0.001	1.508	1.324–1.717	<0.001	2.808	1.445–5.455	0.002
Undifferentiated	1.743	1.410–2.153	<0.001	1.674	1.348–2.079	<0.001	3.746	1.299–10.801	0.015
Linits plastica									
Yes	1			1			1		
No/unknown	0.551	0.358–0.847	0.007	0.581	0.349–0.967	0.037	0.491	0.219–1.100	0.084
Signet ring cell carcinoma									
Yes	1			1			1		
No/unknown	0.865	0.801–0.935	<0.001	0.867	0.794–0.947	0.001	0.867	0.739–1.018	0.082
AJCC TNM, 7^th^ed									
I	1			1			1		
II	2.010	1.860–2.173	<0.001	2.103	1.939–2.280	<0.001	2.427	1.691–3.483	<0.001
III	3.380	3.110–3.674	<0.001	3.255	2.970–3.567	<0.001	6.351	4.559–8.848	<0.001
IV	5.063	4.539–5.648	<0.001	5.009	4.471–5.611	<0.001	20.111	12.326–32.813	<0.001
Lymphadenectomy with at least 15 lymph nodes									
Yes	1			1			1		
No/unknown	1.265	1.200–1.334	<0.001	1.273	1.202–1.348	<0.001	1.203	1.036–1.398	0.016
Neo/adjuvant Chemotherapy^a^									
Yes	1			1			1		
No/unknown	1.056	1.036–1.433	0.013	1.245	1.145–1.354	<0.001	1.098	1.028–1.252	0.042
Neo/adjuvant Radiation^a^									
Yes	1			1			1		
No/unknown	1.207	1.122–1.297	<0.001	1.027	0.953–1.108	0.485	0.834	0.620–1.122	0.230

Adjusted factors: Region, Race, Age, Sex, Year, Grade, Linits plastica, Signet ring cell carcinoma, AJCC TNM 7^th^ed, Surgery, Lymphadenectomy with at least 15 lymph nodes, Neo/adjuvant Chemotherapy, and Neo/adjuvant Radiation.

^a^Neo/adjuvant Chemotherapy: Neoadjuvant chemotherapy OR/AND adjuvant chemotherapy.

Neo/adjuvant Radiation: Neoadjuvant radiation OR/AND adjuvant radiation.

### 3.3 Unadjusted and adjusted survival analysis in different races

The univariate analysis found poor prognosis in Black ethnicity (HR = 4.538, 95% CI: 4.266–4.827, *p *< 0.001) and White ethnicity (HR = 3.716, 95% CI: 3.549–3.890, *p *< 0.001) compared to Chinese PGC patients. This survival outcomes was in accordance with PGC patients only in the US region (Black: HR = 1.431, 95% CI: 1.284–1.596, *p *< 0.001; White: HR = 1.73, 95% CI: 1.173–1.298, *p* = 0.002). To avoid the bias of year and TNM stage, we performed the detailed Kaplan‐Meier survival curves of PGC patients in different races (Figures 3, 4, 5, 6, right column), and showed survival benefit in Chinese PGC group (all *p *< 0.05).

After controlling for confounding variables (Table 3), racial factors were independently associated with poor survival included Black ethnicity (HR = 1.376, 95% CI: 1.066–1.7760, *p* = 0.014) and White ethnicity (HR = 1.262, 95% CI: 1.005–1.583, *p* = 0.045) when compared to Chinese ethnicity in total PGC patients. Even in the same region for only in the US group, Chinese PGC patients also showed better prognosis (Black: HR = 1.403, 95% CI: 1.087–1.811, *p* = 0.009; White: HR = 1.274, 95% CI: 1.016–1.599, *p* = 0.036).

### 3.4 DEGS analysis for gastric cancer patients

**FIGURE 7:**
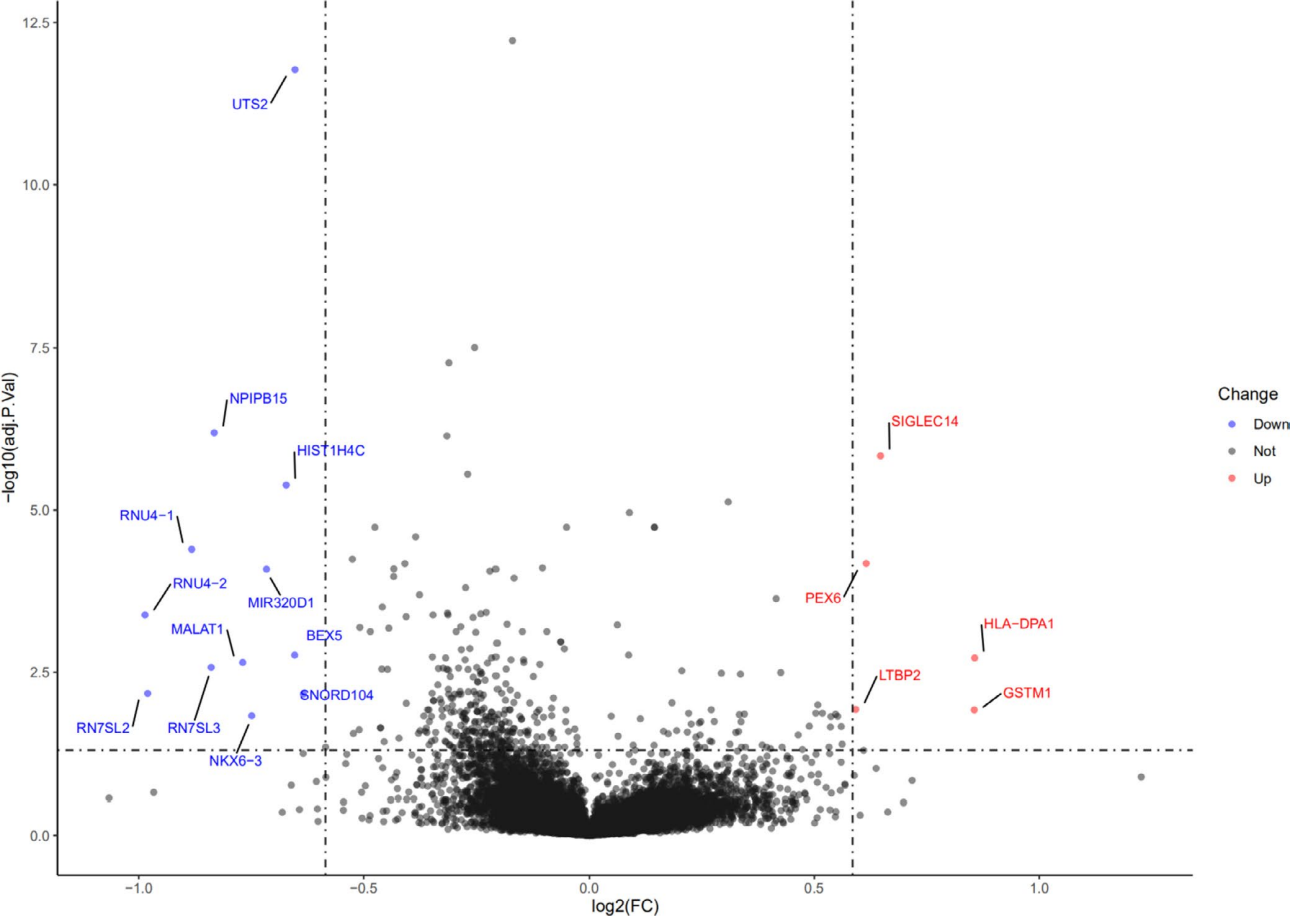
Differentially expressed gene (DEG) analysis of gastric cancer patients from TCGA database (Asian vs. White)

To analyze genetic differences among different races, we performed DEGs analysis for gastric cancer patients from TCGA database (Figure 7). After selecting patients with certain information (race, survival status, and biological information), 236 patients with White ethnicity and 74 patients with Asian ethnicity were included. Compared to Asian PGC patients, some genes (*UTS2*, *NPIPB15*, *HIST1H4C*, *RNU4*‐*1*, *RNU4*‐*2*, *MIR320D1*, *MALAT1*, *BEX5*, *RN7SL2*, *RN7SL3*, *NKXL3*, *SNQRD104*) were down‐regulated in White patients, while *SIGLEC14*, *PEX6*, *HLA*‐*DPA1*, *LTBP2*, and *GSTM1* were up‐regulated.

## 4 DISCUSSION

In our study, we found that the percentage of PGC was rising among total gastric cancer patients from 1996–2016, not only in western counties (SEER database, 29.42% to 36.88%) but also in China (National Cancer Center database, 13.03% to 32.53%). Thus, it is no surprise that so many studies have focused on the PGC in recent times. Finally, 40973 PGC patients were enrolled and the clinicopathological characteristics of PGC patients in the US patients presented differently with PGC patients in China. More importantly, we demonstrated the importance of regional influences as well as the racial features on prognosis for PGC patients from two high‐volume database SEER and China National Cancer Center.

Our findings are consistent with some previous studies—namely, that China group was associated with a better prognosis than the US patients, though the previous studies focused on total gastric cancer rather than only PGC patients.^5^, ^14^, ^15^, ^16^, ^17^ In addition to differences in survival, there are differences in the type of treatment received in different regions. This is more prominent in the management and subsequent outcomes of gastric cancer, one of the most common cancers in the world. Our research investigated PGC patients revealing higher rate of gastrectomy and adequate lymphadenectomy with at least 15 lymph nodes in China region, while curative surgical resection is the gold standard treatment for resectable gastric cancer. As we all know, D2 lymphadenectomy is popular in Asian area while the majority of patients in the US undergo D1 lymphadenectomy.^18^ A previous studied indicated that the median number of lymph nodes retrieved for D1 lymphadenectomy was 13 and for D2 lymphadenectomy was 17.^19^ In our study, we observed that adequate lymphadenectomy with at least 15 lymph nodes was an independent factor for survival in both China and the US group for PGC patients with gastrectomy. These treatment differences may be partly contributed to the survival distinction from populations in different countries.

Furthermore, PGC patients in Western counties were more likely diagnosed with later TNM stage and distant metastasis as studies reported.^4^, ^7^ These may be due to the cancer screening and early detection programs (including cancers of the esophagus, stomach, etc.) which have expanded to 31 provinces as early as 2015 year in China.^20^ When considering disease presentation, China group is more likely to be younger at initial diagnosis than the US group. However, age is an interesting prognostic factor — middle year group (55–64 year) was associated with poor survival outcomes in the US but favorable survival in China when compared to those younger than 45 years. These different patterns of PGC in the East and West are so apparent that many have suggested inherent differences in biologic behavior, such as race/ethnicity itself.

With respect to race itself, this cohort demonstrated that Black and White ethnicity are independently associated with mortality of PGC patients in multivariate analysis when compared to Chinese ethnicity. In our further analysis for only the US group, Chinese ethnicity also had better survival than those patients in White and Black ethnicity. Li et al^2^ have summarized the known protein of different genes in different races of gastric cancer patients based on the published studies. They demonstrated that *GYG2P1*, *RPS4Y1*, *TXLNG*, and *EIF1AX* genes were highly expressed in White population, while *DNAJC5*, *HDAC10*, *NEO1*, and *SMG5* highly expressed in Black man.^2^ In addition, *TMSB4Y*, *UTY*, *ZFY*, and *ZNF787* were significantly associated with Asian patients. Theuer et al ^21^ demonstrated that normal E‐cadherin expression was more common in Japanese intestinal‐type gastric cancer whereas c‐erbB2 expression was higher in American gastric cancers. In our study, we showed some genes differentially expressed between Asian and White population. *LTBP2*, highly expressed in White race, was associated with migration and invasion of gastric cancer cells and predicts poor outcome of patients with gastric cancer.^22^ Above all, the race/ethnicity itself is an indeed important prognostic factor for PGC patients.

In addition, we demonstrated that year was an independent prognostic factor for PGC patients in both China and the US groups. This relative survival improved steadily over time for proximal gastric cancer, indicating an improvement in the quality of clinical services for gastric cancer patients, such as improved access to primary healthcare, greater availability of diagnostic facilities, improved effectiveness of multimodal treatment in recent years.^20^, ^23^ In addition, gastric cancer surgery has been advancing in exploration: how to achieve the optimal extent of lymphadenectomy; and the rapid digital technology development of screen‐based intervention techniques that have led to minimally invasive interventions such as endoscopic mucosal resections for early gastric cancer and laparoscopic and robotic gastrectomy techniques for early and locally advanced gastric cancer.^24^ These all made greatly achievement the survival of gastric cancer.

Our study has numerous strengths. First, two large population databases—SEER and China National Cancer Center database, were utilized to demonstrated the importance of regional influences as well as the racial features for PGC patients, leading to an adequately powered study. Secondly, we discussed not only regional factors like treatment but also race/ethnicity itself in different populations of PGC patients thus provided a better understanding of these disparities. Lastly, we were able to adjust in our multivariable model for the most important prognostic factors in gastric cancer—specifically AJCC 7^th^ TNM stage, surgery status, lymph nodes, and neo/adjuvant therapy—and therefore, controlled for the possibility that the decreased mortality amongst China is solely due to an earlier stage of diagnosis. Despite all this, we acknowledge limitations of our study. SEER database does not include all regional prognostic indictors, like environmental exposures and lifestyle factors (e.g., smoking, drinking, Hp infection and BMI), which may influence the prognosis of PGC patients. In addition, China National Cancer Center was a single institution, so the results might not represent the whole Chinese population, although the database was one of the biggest gastric cancer database in China. Third, due to the limitation of variables in China National Cancer Center database or the SEER database, some important factors, such as morbidity, mortality, surgical margins, Karnofsky or ECOG status, Charleson‐Deyo comorbidity score, type of surgery, are not evaluated in this study. Neo/adjuvant chemotherapy means neoadjuvant chemotherapy or/and adjuvant chemotherapy in this study, because we just got the information of chemotherapy yes or not from SEER database rather than neoadjuvant and adjuvant chemotherapy. Fourth, a period of 20 years was examined because of differences in treatment and diet, and potentially variable environmental factors. These factors could affect the accuracy of the results.

In conclusion, we demonstrated the different survival outcomes of PGC patients in different regions or races from two high‐volume database SEER and China National Cancer Center database. These survival differences are likely influenced by a number of factors (e.g. access to screening, quality of gastrectomy, neo/adjuvant therapy, and biological genes itself) and a better understanding of these disparities could lead to interventions that may help to abolish these disparities. Studies are warranted to further investigate the disparities of PGC patients in molecular mechanism.

## CONFLICTS OF INTEREST

All authors have completed the ICMJE uniform disclosure form (available at http://dx.doi. org/10.21037/atm‐20‐2554a). The authors have no conflicts of interest to declare.

## AUTHORS’ CONTRIBUTIONS

(I) Conception and design: Yingtai Chen; (II) Administrative support: Dongbing Zhao, Yingtai Chen; (III) Provision of study materials or patients: Lulu Zhao, Yingtai Chen; (IV) Collection and assembly of data: Lulu Zhao, Penghui Niu; (V) Data analysis and interpretation: Lulu Zhao, Penghui Niu; (VI) Manuscript writing: All authors; (VII) Final approval of manuscript: All authors.

## ETHICAL STATEMENT

The authors are accountable for all aspects of the work in ensuring that questions related to the accuracy or integrity of any part of the work are appropriately investigated and resolved. This study was approved by the ethics committee of National Cancer Center/National Clinical Research Center for Cancer/Cancer Hospital, Chinese Academy of Medical Sciences and Peking Union Medical College (No. 17‐156/1412).

## Data Availability

The data used to support the fndings of this study are included within the article in Tables.
